# The Carotid Web: A Rare Abnormality of the Intimal Layer Causing Ischemic Stroke in a Young Female Patient

**DOI:** 10.7759/cureus.71786

**Published:** 2024-10-18

**Authors:** Konstantinos G Seretis, Nikolaos Giannakopoulos, Polixeni Stasinaki, Alexandra Souli-Bakaloglou, Theofanis Papas

**Affiliations:** 1 Department of Vascular Surgery, Korgialenio-Benakio Hellenic Red Cross Hospital, Athens, GRC; 2 Department of Neurology, Korgialenio-Benakio Hellenic Red Cross Hospital, Athens, GRC

**Keywords:** carotid artery disease, carotid intima-media, carotid web, cryptogenic strokes, extracranial fibromuscular dysplasia

## Abstract

The carotid web represents a specific type of fibromuscular dysplasia that primarily affects the intimal layer and is considered a high-risk factor for cryptogenic ischemic stroke. There is still debate regarding the ideal diagnostic imaging for carotid webs. Computed tomography angiography (CTA) is the preferred method in most studies; however, digital subtraction angiography (DSA) has been proven to offer great-quality images for diagnosing and evaluating the carotid web. Surgical treatment of carotid web is essential in order to avoid recurrent attacks of ischemic stroke. Carotid endarterectomy (CEA) and carotid artery stenting (CAS) are both indicated for the treatment of carotid web; on the other hand, the efficacy of conservative treatment is still debated.

We present a case of ischemic stroke in a young female patient that was attributed to a carotid web and was treated successfully with CAS of the right internal carotid artery.

## Introduction

The carotid web is a thin layer of proliferative intimal tissue that spreads into the vessel lumen from the arterial wall. Unlike conventional fibromuscular dysplasia, which mainly involves the media, the carotid web appears to be a distinct kind of fibromuscular dysplasia that predominantly involves the intimal layer. The carotid web was first described in 1968 by Rainer et al. [1] and is considered a high-risk factor for ischemic stroke of unknown etiology [2-4], with a reported incidence of 9.4% among patients with cryptogenic ischemic stroke [5].

The exact mechanism by which the carotid web causes an ischemic stroke is still unknown. According to Choi et al. [6], the abnormal protrusion into the vessel lumen is thought to disturb the intraluminal flow, resulting in a slow or turbulent flow that promotes the development of mural thrombus, which in turn is the cause of stroke or stroke recurrence.

There is no clear consensus on the ideal imaging method, and research is still needed to establish guidelines for the definite diagnostic approach of carotid webs [7]. Treatment strategies include primarily surgical procedures, carotid endarterectomy (CEA) and carotid artery stenting (CAS), since the efficacy of conservative treatment is still debated [8,9].

## Case presentation

**Figure 1:**
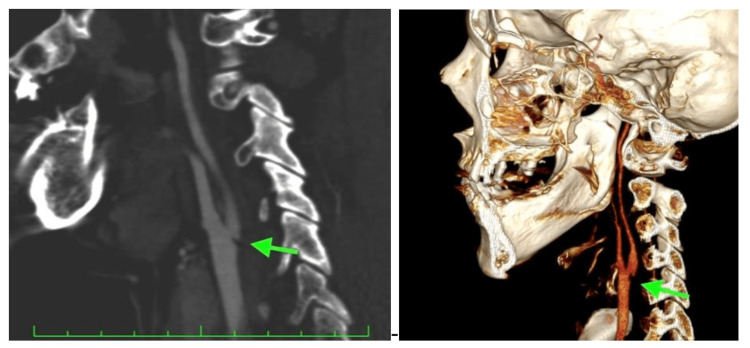
Computed tomography angiography (CTA) reveals a localized membrane-filling defect (green arrow)

Our patient, a 42-year-old female smoker without any past medical history, visited the emergency room (ER) of our hospital for a referred left-side motor deficit that started two hours prior to her visit. She was examined by the attending neurologist in the ER and was found to have mild left-side hemiparesis and sensory loss of her left extremities, and mild dysarthria (NIHSS: 6). Brain CT was normal, and CTA of the vessels responsible for brain circulation revealed a carotid web of the right internal carotid artery (Figure 1).

A cardiologic examination didn't reveal any acute pathologic findings (normal ECG, normal transthoracic echocardiography, ejection fraction (EF) 65%), and after the primary evaluation, she was admitted to the neurological department. Immediately following her admission, she received treatment with systemic thrombolysis (63 mg of alteplase in total). Transoesophageal echocardiography that was performed the next day in order to complete her evaluation was found normal, and our patient was set on dual antiplatelet treatment (DAPT) and statin therapy. 

**Figure 2:**
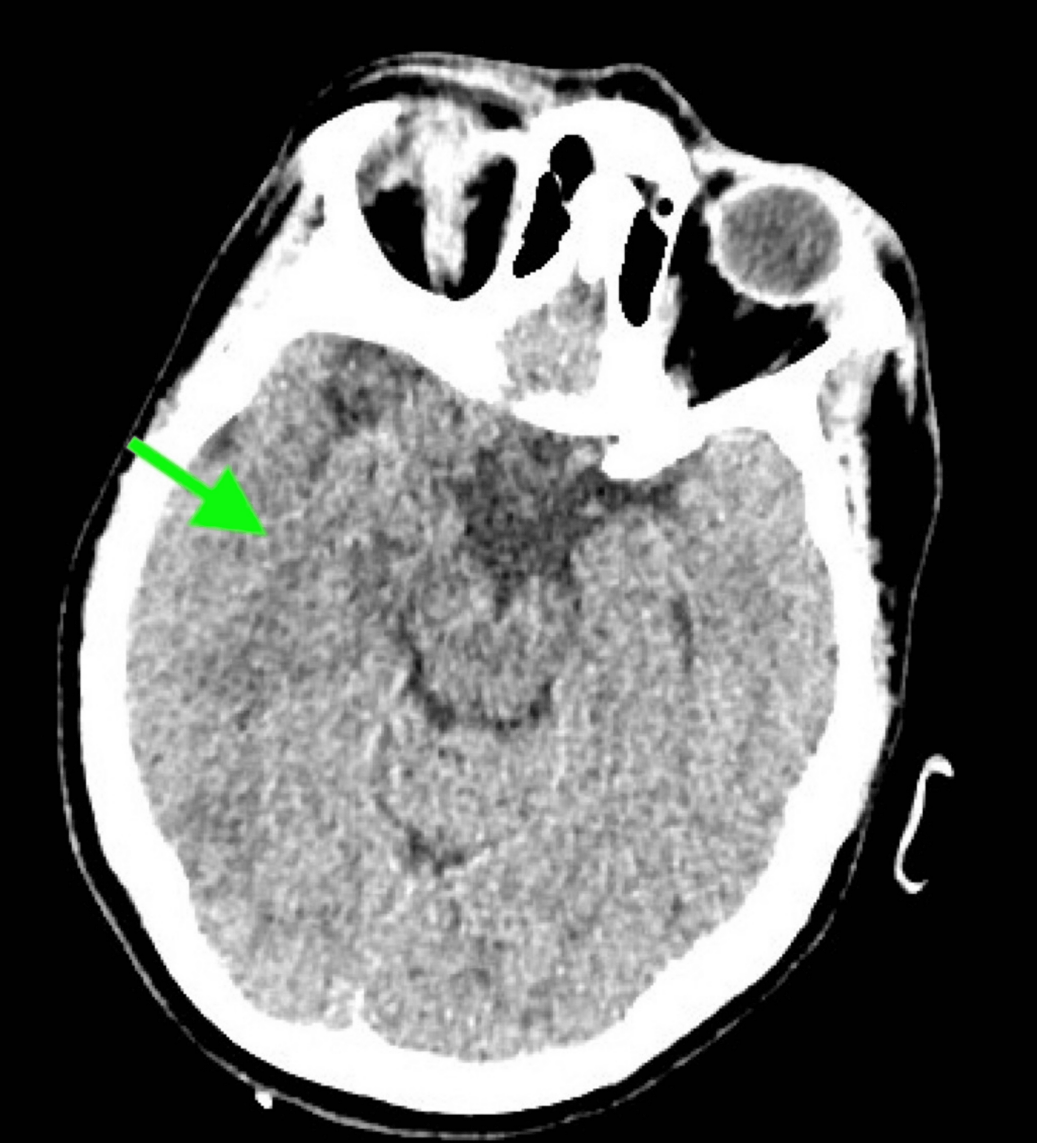
Brain CT showing a hypodense region at the level of posterior limb (posterior crus) of the internal capsule and the lentiform nucleus (green arrow)

Brain CT was repeated 24 hours after her admission and revealed a hypodense region at the level of the posterior limb (posterior crus) of the internal capsule and the lentiform nucleus (Figure 2).

**Figure 3:**
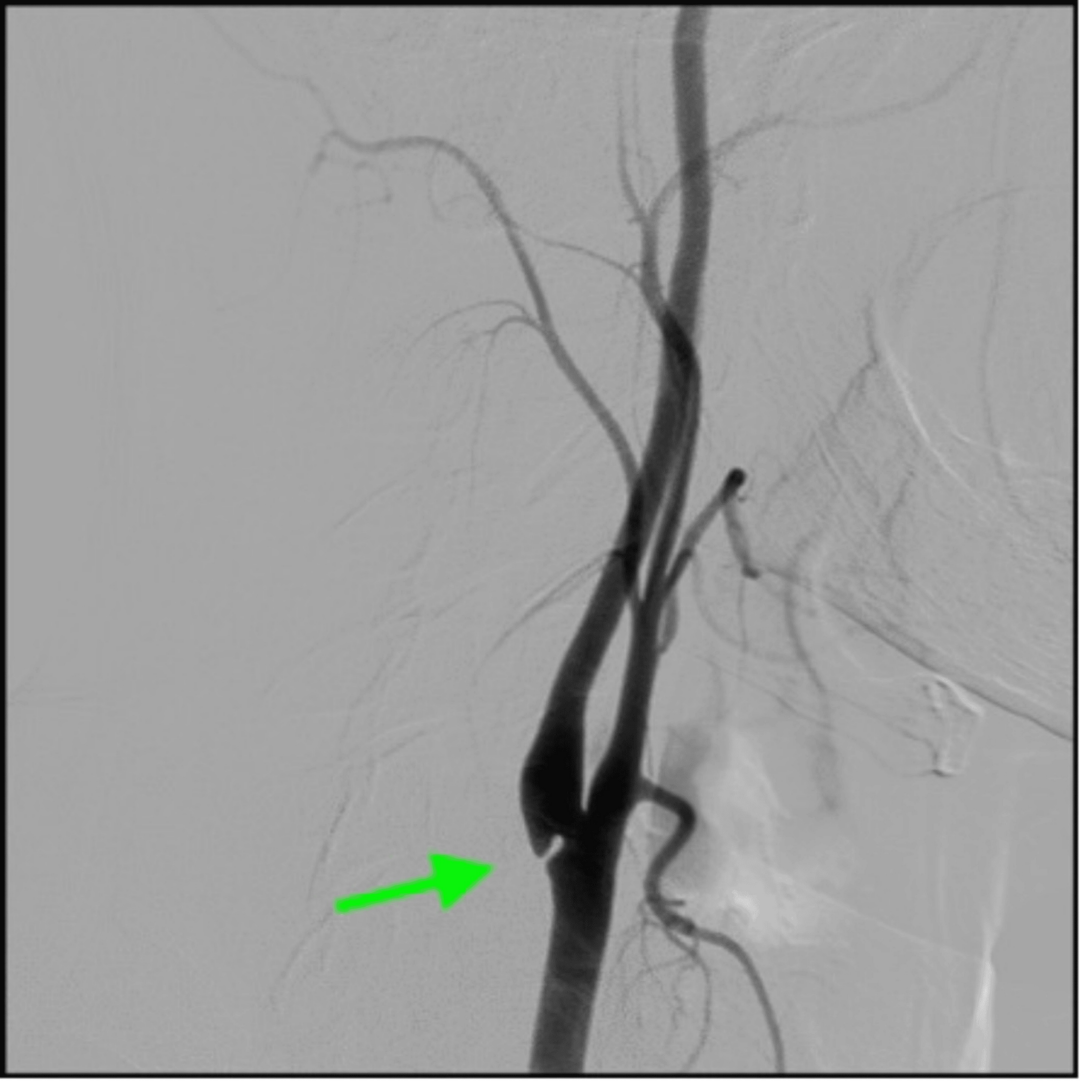
Carotid digital subtraction angiography (DSA) shows a filling defect in the posterior wall of the carotid bulb (arrow)

Based on those findings, the definite diagnosis was set as an ischemic stroke caused by a carotid web of the right internal carotid artery. After consultation with the vascular surgery team, it was decided to treat our patient with carotid artery stenting (CAS) in order to avoid recurrent ischemic stroke attacks in the future. Digital subtraction angiography (DSA), which was carried out at the beginning of the procedure, confirmed the definite diagnosis (Figure 3).

**Figure 4:**
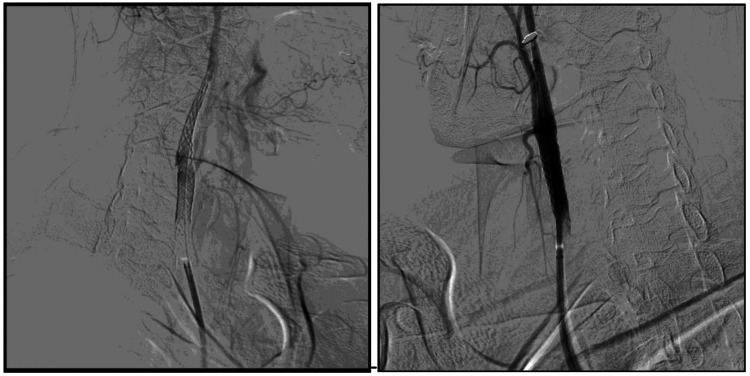
Successful deployment of the carotid stent in the right internal carotid artery

The CAS that followed was performed uneventfully (Figure 4).

Our patient was discharged two days after the procedure, toward the end of the acute phase of the initial event, with only a mild motor deficit (NIHSS: 2), and reached full recovery one month after the initial event.

## Discussion

Although the carotid web is a known cause of ischemic stroke in young patients <65 years of age, there is still debate about the ideal imaging method for the diagnosis of the carotid web. In most studies, CTA has been recommended as the gold standard for the diagnosis of the carotid web, and usually, the sagittal plane shows a localized membrane-filling defect that is diagnostic for the carotid web [10]. However, other imaging modalities are preferred by many centers which appear to be effective in identifying and assessing the carotid web. Digital subtraction angiography (DSA) offers images of great quality, depicting a linear filling defect along the carotid artery wall or a shelf-like filling defect located in the internal carotid artery bulb, both of which positively establish the diagnosis of the carotid web [11]. Similar carotid web detection rates have been reported when comparing DSA and CTA, but CTA offers multiple advantages, such as its high resolution, short scan duration, and the ability to construct related vessels quickly. The carotid web may also appear as a film-like structure during routine ultrasound imaging in the hands of an experienced ultrasound specialist [12]; however, diagnosis can easily be missed in the hands of a less experienced ultra-sonographer or because of the overload of the ER ultrasound department in a high-volume hospital.

Carotid endarterectomy (CEA) and carotid artery stenting (CAS) are both indicated for the treatment of the carotid web, and both have shown good results. Several studies showed that patients with surgically treated carotid webs had no recurrent strokes during the period of follow-up [13-16]. Conservative treatment with anticoagulation and antiplatelet therapies has been proposed as an alternative mode of treatment but might not be enough to avoid recurrent attacks of ischemic stroke [17], and the efficacy of conservative treatment is still debated. 

No treatment guidelines have been made regarding the optimal management of symptomatic patients with carotid webs, although the AHA identified it as an area warranting future research [18]. The latest European Society for Vascular Surgery (ESVS) 2023 Clinical Practice Guidelines on the Management of Atherosclerotic Carotid and Vertebral Artery Disease included a new recommendation for symptomatic patients with a carotid web in whom no other cause for stroke can be identified after detailed neurovascular work up, proposing carotid endarterectomy or carotid artery stenting to be considered in order to prevent recurrent stroke [19].

## Conclusions

Due to its rare occurrence, the carotid web has primarily been documented in case reports and case series, which has left the scientific community with limited knowledge about it. As a result, it is frequently underdiagnosed in clinical practice and has a low detection rate. CTA has been the recommended method for the diagnosis of the carotid web in most studies; however, other imaging methods have been proven to be very good for diagnosing and evaluating the carotid web, and the combination of multiple imaging modalities can provide useful information for the definite diagnosis of the carotid web.

There are still many areas that have to be elucidated, such as the pathogenesis of the carotid web, which still remains unclear, the correlation between the carotid web and cryptogenic ischemic stroke, which requires more data and further analysis, and the carotid web treatment strategy guidelines which are still a matter of debate. Future prospective research is needed to establish the optimal medical or interventional management of symptomatic carotid webs.
